# The in vitro estrogenic activity of the crude drugs found in Japanese herbal medicines prescribed for menopausal syndrome was enhanced by combining them

**DOI:** 10.1186/s12906-018-2170-4

**Published:** 2018-03-23

**Authors:** Zeyun Wang, Seiji Kanda, Takaki Shimono, Dambajamts Enkh-Undraa, Toshimasa Nishiyama

**Affiliations:** Department of Public Health, Kansai Medical University, 2-5-1, Shin-machi, Hirakata-shi, Osaka 573-1010 Japan

**Keywords:** Estrogenic activity, Japanese herbal medicines, Metabolism, Synergistic effect

## Abstract

**Background:**

Japanese herbal medicines can be used as alternatives to estrogen therapy and are sometimes prescribed for menopausal syndrome because they have fewer side effects and are associated with better compliance than estrogen therapy, but little is known about the pharmacological mechanisms of such treatments. This study aimed to explore the mechanisms responsible for the estrogen-like effects of five widely prescribed Japanese herbal medicines *(unkeito*, *kamishoyosan*, *nyoshinsan*, *keishibukuryogan*, and *tokishakuyakusan*).

**Methods:**

We evaluated the estrogenic activity of these five Japanese herbal medicines and their metabolites using an estrogen receptor (ER)-dependent cell proliferation bioassay and an ER-dependent reporter assay. We also investigated the estrogenic activity of the crude drugs within the medicines and attempted to detect inter-crude drug synergistic effects using the ER-dependent reporter assay.

**Results:**

We found that *unkeito*, *kamishoyosan*, and *nyoshinsan* exhibited estrogenic activity, and they displayed stronger estrogenic activity after being metabolized. Then, we focused on investigating the estrogenic activity of the crude drugs present within *unkeito*. We found that glycyrrhizae radix, cinnamomi cortex, evodiae fructus, and zingiberis rhizoma demonstrated ERβ-dependent estrogenic activity. The combined use of evodiae fructus and glycyrrhizae radix, or evodiae fructus and cinnamomi cortex produced synergistic ERβ-dependent estrogenic activity.

**Conclusion:**

It was suggested that *unkeito*, *kamishoyosan*, and *nyoshinsan* exert estrogenic activity, and hence, might be useful for treating menopausal syndrome. Furthermore, synergistic estrogenic effects were detected between some of the crude drugs present within *unkeito*.

## Background

The menopause is a biological stage in a woman’s life when her ovarian function diminishes and eventually ceases. It involves the cessation of both egg maturation and sex hormone (principally estrogen and progesterone) secretion. Many women experience menopausal syndrome, which can cause hot flushes, night sweats, menstrual irregularities, vaginal dryness, etc., during the menopause and perimenopause [1–3]. Estrogen therapy is widely used to treat menopausal syndrome. The biological activity of estrogen is mediated by its binding to estrogen receptors (ER); i.e., ERα and ERβ, which are encoded by different genes and exhibit tissue-type- and cell-type-specific expression. The activation of each subtype leads to different effects. In addition, ERα is associated with certain types of estrogen-dependent tumors, such as breast [4], endometrial [5], and ovarian cancer [6]. On the other hand, it has been found that ERβ expression can inhibit motility and cell invasion in breast [7], prostate [8], and ovarian cancer cells [9]. For example, estrogen increased the proliferation of breast cancer cells in the presence of ERα, but this was inhibited in the presence of ERβ [10]. Therefore, while estrogen therapy might have rapid effects on menopausal syndrome, it might also increase the risk of estrogen-dependent tumors.

Herbal medicines are derived from various plants and have been prescribed to treat many diseases for thousands of years. It was previously reported that phytoestrogens, such as daidzein and daidzin, can be found in puerariae radix [11], and the glabridin present in licorice roots was suggested to be a useful treatment for post-menopausal women because of its estrogenic activity [12–14]. Herbal medicines have recently been used as an alternative to estrogen therapy for menopausal syndrome, but little is known about the pharmacological activity of such medicines. In this study, we evaluated the estrogenic activity of five widely used Japanese herbal medicines (*unkeito [UKT]*, *kamishoyosan [KSS]*, *nyoshinsan [NSS]*, *keishibukuryogan [KBG]*, and *tokishakuyakusan [TSS]*), in order to explain their pharmacological activity against menopausal symptoms.

We examined the estrogenic activity of these five Japanese herbal medicines using a combination of an ER-dependent cell-MtT/Se cell proliferation bioassay and a yeast two-hybrid reporter assay. The MtT/Se cell line was established from rat pituitary cells [15], and both ERα and ERβ are expressed in the cytoplasm around the nuclei of these cells [16]. It was reported that this cell line responds well (and dose-dependently) to 17β-estradiol (E2) stimulation, and the E2-induced proliferation of MtT/Se cells was dose-dependently inhibited by tamoxifen [15]; therefore, an assay based on MtT/Se cell proliferation was developed to evaluate the estrogenic activity of test substances [16]. In addition, a yeast-based estrogen screening test based on the yeast two-hybrid system was used to clarify the estrogenic activity of Japanese herbal medicines. This system was constructed by inserting the ligand-binding domain (LBD) of the human estrogen receptor and its coactivator into yeast cells, which allowed the specific binding of ligands to the ER-LBD. Estrogenic activity was then quantitatively evaluated based on the expression level of a reporter gene encoding the β-galactosidase enzyme [17].

Like all drugs, the chemical structures and pharmacological activity of Japanese herbal medicines might be altered by the enzymatic systems found in living organisms. For example, the estrogenic activity of the phytoestrogen-rich plant Pueraria mirifica has been found to increase significantly after being metabolized [18]. Therefore, the metabolic modulation of estrogenic activity must be considered in any assessment of the impact of Japanese herbal medicines. In this study, we also investigated the estrogenic activity of Japanese herbal medicines after they had been metabolized by the rat liver S9 fraction.

Herbal medicines are composed of a mixture of several crude drugs. In order to study the pharmacological activity of herbal medicines, we first need to confirm which of the crude drugs present within them are responsible for such activity. We assessed the estrogenic activity of the crude drugs using the same protocol as was used to study the Japanese herbal medicines. We also conducted an experiment to detect inter-crude drug synergistic effects.

## Methods

### Medicines and extraction

Japanese herbal medicines; i.e., *UKT* (unkei decoction, TJ-106), *KSS* (kamishoyo powder) (TJ-24), *NSS* (nyoshin powder, TJ-67), *KBG* (keishibukuryo pills, TJ-25), and *TSS* (tokishakuyaku powder, TJ-23), were obtained from Tsumura Corporation (Tokyo, Japan) in the form of dried powder extracts.

Each herbal medicine extract powder was manufactured as an aqueous extract containing processed raw materials in the ratios shown in Table 1. The qualities of these raw materials were tested as set out in the Japanese Pharmacopoeia. Each herbal medicine was extracted with purified water at 95°C for 1 h. The extract solution was separated from the non-soluble waste and concentrated by removing water under reduced pressure. Spray-drying was used to produce a dried extract powder. The same extraction procedure was applied to each crude drug.

**Table 1:** The mass ratio of crude drugs in the Japanese herbal medicines

herbal	simplified form	Kamishoyosan	Keishibukuryogan	Nyoshinsan	Tokishakuyakusan	Unkeito
		KSS	KBG	NSS	TSS	UKT
alismatis rhizoma	ar				4	
angelicae acutilobae radix	aar	3		3	3	3
arecae semen	as			2		
asini corii collas	acc					2
atractylodis lanceae rhizoma	alr	3		3	4	
bupleuri radix	br	3				
caryophylli flos	cf			1		
cinnamomi cortex	cc		3	2		2
cnidii rhizoma	cr			3	3	2
coptidis rhizoma	ctr			1		
cyperi rhizoma	cer			3		
evodiae fructus	ef					1
gardeniae fructus	gf	2				
ginseng radix	gra			2		2
glycyrrhizae radix	gr	1.5		1		2
menthae herba	mh	1				
moutan cortex	mc	2	3			2
ophiopogonis radix	or					4
paeoniae radix	pr	3	3		4	2
persicae semen	ps		3			
pinelliae tuber	pt					4
poria sclerotium	pst	3	3		4	
saussureae radix	sr			1		
scutellariae radix	scr			2		
zingiberis rhizoma	zr	1				1

The mass ratio of crude drugs in the Japanese herbal medicines was provided to us by Tsumura Corporation. In order to simplify the name of herbal medicines and crude drugs in text, we made the simplified forms in the table

The active ingredient of each powdered medicine was extracted by mixing 1 g of powder with 10 ml of 99.5% ethanol and rotating the mixture for 4 h in our laboratory. The mixture was then filtered through a 0.45-μm filter membrane. The solvent was evaporated to dryness at room temperature in a vacuum, and the residue was dissolved in dimethyl sulfoxide (DMSO) (Wako Chemical, Japan). The concentration of each extract was adjusted with DMSO as required for each assay.

### Metabolic activation

In this study, in vitro metabolic activation via the addition of the rat liver S9 fraction (Sigma Aldrich, Saint Louis, USA) was used to metabolize the examined Japanese herbal medicines. Metabolic activation was induced using an S9 mixture containing 0.5 mg/ml of the S9 fraction in 100 mM potassium phosphate buffer (pH 7.4), 3.3 mM MgCl_2_ (Wako Chemical, Japan), and an NADPH-generating system [1.3 mM NADP (Sigma Aldrich, USA), 3.3 mM glucose 6-phosphate (Oriental Yeast, Tokyo, Japan), and 0.4 units/ml of glucose-6-phosate dehydrogenase (Oriental Yeast, Tokyo, Japan)]. Each 200-μl sample of Japanese herbal medicine dissolved in DMSO was incubated with 800 μl of the S9 mixture at 37°C for 1 h or with 800 μl of the negative mixture, which was composed of the abovementioned S9 mixture minus the S9 fraction, as a negative control. The metabolized Japanese herbal medicines were stored at − 80°C until use.

### Cell culture and maintenance of the MtT/se cells

Rat mammotropic pituitary tumor cells, MtT/Se cells, were donated by the Riken Bioresource Center cell bank in Japan. The MtT/Se cells were routinely cultured in Dulbecco’s modified Eagle’s medium (DMEM)/HamF12 (1∶1) medium (Nacalai Tesque, Japan) containing 2.5% fetal bovine serum (FBS) (HyClone, USA), 10% horse serum (HS) (Invitrogen, USA), and 1 × 10^− 9^ M of estradiol (E2) (Wako Chemical, Japan). The cells were incubated at 37°C in a humidified atmosphere of 5% CO_2_ and 95% air.

### ER-dependent cell proliferation bioassay

Before the cell proliferation bioassay, the MtT/Se cells were maintained for 4 days in routine medium from which E2 had been removed, as described above. In the proliferation assay, the MtT/Se cells were placed in phenol red-free DMEM/HamF12 (1:1) medium (Nacalai Tesque, Japan) containing 2.5% charcoal dextran-treated FBS (HyClone, USA) and 10% HS at a density of 1 × 10^5^ cells/ml, before being seeded at 95 μl/well in 96-well microplates. Then, the cells in each well were incubated with 5 μl of each test substance for 6 days. In order to clarify whether the herbal medicines induced cell proliferation through ER-mediated mechanisms or other general mitogenic pathways, ICI-182,780, an ER antagonist (Abcam, UK), was added as a competitor. Cells that were incubated with DMSO alone were used as a control, and the final concentration of DMSO in all wells was 0.1%. Six days later, cell proliferation was measured with a modified MTT assay based on WST-8, a highly water-soluble tetrazolium salt (Dojindo, Japan). The amount of formazan dye formed by metabolically active cells was quantified by measuring the absorbance of each well at 450 nm. The estrogenic activity of each test substance was evaluated based on the cell proliferation that occurred in its presence, which was calculated by comparing the associated A_450nm_ value with the A_450nm_ value of the control, DMSO.

Cell proliferation (%) = (A_450nm test_ / A_450nm DMSO_) × 100%.

### ER-dependent reporter assay (yeast two-hybrid assay)

In this study, we used human ERα (hERα) and hERβ yeast cells carrying the LBD of hERα or hERβ and the coactivator transcriptional intermediary factor 2 (TIF2) (the cells had been transfected with two expression plasmids, pGBT9-ERLBD and pGAD424-TIF2) [19].

The yeast cells were pre-incubated and shaken overnight at 30°C in selective medium (SD medium lacking tryptophan and leucine). A 100-μl sample of the overnight yeast cell culture was added to 400 μl of fresh medium containing 5 μl of one of the test substances. After being incubated for 4 h at 30°C, the yeast cells were collected by centrifugation and resuspended in 250 μl Z-buffer [0.1 M sodium phosphate (Wako Chemical, Japan), 10 mM KCl (Wako Chemical, Japan), 1 mM MgSO_4_ (Wako Chemical, Japan)]. Then, 150 μl of the cell suspension were added to a 96-well microplate, and the cell density of each well was measured based on its A_600nm_ value. Next, the cell walls of the remaining 100 μl of yeast cells were digested enzymatically by incubating them with 100 μl of 2 mg/ml Zymolyase 20 T (Nacalai Tesque, Japan) at 37°C for 30 min. Then, the 200-μl lysate was mixed with 40 μl of 4 mg/ml o-nitrophenyl-β-D-galactopyranoside (ONPG) (Sigma Aldrich, USA) and incubated at 30°C for 1 h, before the enzymatic reaction was stopped by the addition of 100 μl of 1 M Na_2_CO_3_ (Wako Chemical, Japan). Then, the yeast debris was removed by centrifugation, and the absorbance of the supernatant was measured at 450 nm. β-galactosidase activity was calculated using the following equation:

$$ \upbeta -\mathrm{galactosidaseactivity}\left(\mathrm{units}\right)=1000\times {\mathrm{A}}_{450\mathrm{nm}}/\left(60\min \times 0.1\mathrm{ml}\times {\mathrm{A}}_{600\mathrm{nm}}\right) $$

, where A_600nm_ represents the cell density at the start of the assay, A_450nm_ represents the absorbance of the supernatant at the end of enzymatic reaction, 60 min is the duration of the enzymatic reaction, and 0.1 ml is the volume of the yeast cell culture used in the enzymatic reaction.

The ERα estrogenic activity of the tested Japanese herbal medicines was evaluated based on the β-galactosidase activity of hERα yeast cells, and their ERβ-dependent estrogenic activity was assessed based on the β-galactosidase activity of hERβ yeast cells. REC_10_ (the concentration that produced an activity level equivalent to 10% of the maximum activity of E2) was used to evaluate the estrogenic activity of the Japanese herbal medicines.

### Combination study of synergism and synergy quotient calculations

As shown in Table 1, Japanese herbal medicines are composed of various crude drugs, and we tried to identify pairs of crude drugs that had synergistic effects. In the combinatorial study, each pair of crude drugs was added to yeast cells in a 1:1 ratio. The combinatorial study was performed to detect synergistic crude drug effects on ERβ-dependent estrogenic activity compared with the activity seen during the administration of each drug alone.

The synergism quotient (SQ) was calculated by subtracting the baseline values (the β-galactosidase activity values of DMSO) from all treatments and then dividing the activity value of the combined treatment by the sum of the activity values for the individual treatments. A SQ of > 1.0 indicated a synergistic effect [20, 21].

### 3D-high-performance liquid chromatography (HPLC) analysis

In this study, 3D-HPLC analyses of the herbal medicines were performed by Tsumura Corporation to identify each of their components.

The HPLC apparatus consisted of a Shimadzu LC-10A (analysis software: CLASS-M10A ver. 1.64, Tokyo, Japan) equipped with a multiple wavelength detector (wavelength range: 200–400 nm; Shimadzu SPD-M10AVP diode array detector) and an autoinjector (Shimadzu CTO-10 AC). The HPLC conditions were as follows: column, TSK-GEL 80TS octadecylsilyl column (internal diameter: 250 × 4.6 mm; TOSOH, Tokyo, Japan); eluent, (A) 0.05 M AcONH_4_ (pH 3.6), (B) 100% CH_3_CN. A linear gradient of 90% A and 10% B changing over 60 min to 0% A and 100% B was used. Once a B concentration of 100% had been achieved, it was maintained for 20 min (temperature, 40°C; flow rate, 1.0 mL/min).

### Statistical analysis

All experiments were conducted at least three times for each sample, and all data shown represent the mean ± standard deviation (SD). The Kolmogorov-Smirnov test was used to confirm that all of the data for each sample exhibited a normal distribution. All statistical analyses were carried out using the Student’s t-test in SPSS, version 15.0. *P*-values of < 0.05 were considered to be statistically significant.

## Results

The estrogenic activity of Japanese herbal medicines and their metabolites in an ER-dependent cell proliferation bioassay.

**Fig. 1:**
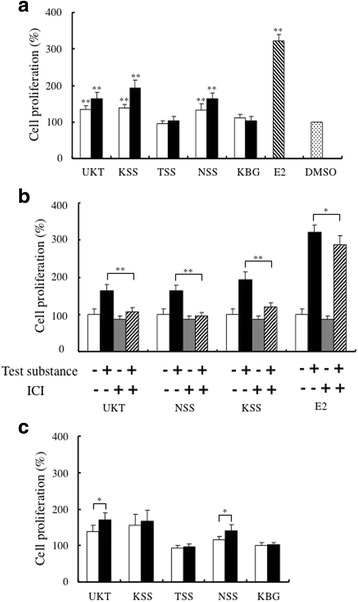
The cell proliferation activity induced by Japanese herbal medicines. **a** MtT/Se cells were incubated with 50 μg/ml (white columns) or 100 μg/ml of each Japanese herbal medicine (black columns) or 10^− 7^ M of E2 for 6 days. Cell proliferative activity is shown as a percentage compared with that induced by DMSO alone in the MTT assay. **significantly increased compared with the activity induced by DMSO, *p* < 0.01. **b** Cells were treated with 10 nM ICI alone or a combination of 10 nM ICI and 100 μg/ml of *UKT*, *KSS*, or *NSS* or 10^− 7^ M E2 for 6 days. White columns: ICI(−)test substance(−); blank columns: ICI(−)test substance(+); gray columns: ICI(+)test substance(−); diagonal columns: ICI(+)test substance(+). **significantly inhibited the induction of cell proliferation compared with herbal medicines or E2 alone, *p* < 0.01; *p < 0.05. **c** The cell proliferation induced by metabolized Japanese herbal medicines. The Japanese herbal medicines were treated with the rat S9 fraction, and the proliferative activity induced by each Japanese herbal medicine (100 μg/ml, black columns) was compared with that induced by the unmetabolized medicine (white columns). *significantly increased compared with the activity of the unmetabolized medicine, *p* < 0.05

The estrogenic activity of each Japanese herbal medicine was evaluated using an MtT/Se cell proliferation assay. The cell proliferation that was induced by E2 plateaued at 10^− 7^ M, and the mean cell proliferation activity level was 322% higher than the activity induced by DMSO. *UKT*, *KSS*, and *NSS* induced significantly increased cell proliferation at final concentrations of 50 μg/ml and 100 μg/ml. However, neither *TSS* nor *KBG* induced significant increases in cell proliferation at concentrations of 50 μg/ml or 100 μg/ml (Fig. 1a). When the cells were treated with 10 nM of ICI, the proliferation induced by *UKT*, *KSS*, *NSS*, and E2 was inhibited (Fig. 1b). These results showed that *UKT*, *KSS*, and *NSS* induced proliferation through ER-mediated mechanisms and not via other general mitogenic pathways. Thus, these three herbal medicines displayed estrogenic activity, and *KSS* exhibited the strongest activity in this bioassay.

In order to study the effects of the examined Japanese herbal medicines after they had been metabolized, we evaluated their estrogenic activity after they had been pre-treated with rat liver S9 extract in vitro. Metabolized *UKT* and *NSS* induced significantly greater cell proliferation than unmetabolized *UKT/NSS* at a final concentration of 100 μg/ml (Fig. 1c). The estrogenic activity of *KSS* also increased after it was treated with S9, but the difference was not significant. These results showed that the estrogenic activity of *UKT* and *NSS* increased about 1.2-fold after they were treated with S9. *TSS* and *KBG* did not show any estrogenic activity before or after they were metabolized.

Estrogenic activity of Japanese herbal medicines and their metabolites in a yeast two-hybrid assay.

**Fig. 2:**
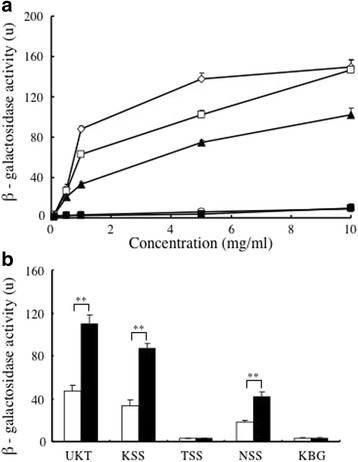
The ERβ-dependent estrogenic activity of the Japanese herbal medicines and the metabolized herbal medicines in the yeast two-hybrid assay. **a** Yeast cells that had been transfected with hERβ were incubated with 0.1 mg/ml to 10 mg/ml of *UKT* (◊), *KSS* (□), *TSS* (○), *NSS* (▲), or *KBG* (■) for 4 h at 30°C. ERβ-dependent estrogenic activity was evaluated based on the β-galactosidase activity measured at 450 nm using ONPG as a substrate. **b** The Japanese herbal medicines (1 mg/ml) were treated with the rat S9 fraction, and the ERβ-dependent estrogenic activity of the metabolized herbal medicines (black columns) was compared with that of the unmetabolized medicines (white columns) using the yeast two-hybrid assay. **significantly increased compared with the activity of the unmetabolized medicine, *p* < 0.01

Next, we evaluated the ERβ-dependent estrogenic activity of the five examined Japanese herbal medicines. We analyzed the ERβ-dependent estrogenic activity of each medicine using a reporter gene-based yeast two-hybrid assay at final concentrations of 0.1 mg/ml to 10 mg/ml (Fig. 2a). Marked β-galactosidase activity was induced by *UKT*, *KSS*, and *NSS*, and the activity increased dose-dependently. These results suggested that *UKT*, *KSS*, and *NSS* exhibited estrogenic activity, which agrees with their estrogenic effects in the MtT/Se cell proliferation assay. On the other hand, *TSS* and *KBG* demonstrated low ERβ-dependent estrogenic activity, even at higher concentrations. In addition, E2 induced ERβ-dependent estrogenic activity at concentrations ranging from 1 × 10^− 4^ M to 1 × 10^− 11^ M, and this activity plateaued at an E2 concentration of 1 × 10^− 6^ M. The REC_10_ values of *UKT*, *KSS*, and *NSS* were 0.7 mg/ml, 2.2 mg/ml, and 4.5 mg/ml, respectively. These results suggested that *UKT*, *KSS*, and *NSS* induced marked ERβ-dependent estrogenic activity. *UKT* had the lowest REC_10_ value of the five examined Japanese herbal medicines, which showed that it produced the strongest ERβ-dependent estrogenic activity. On the other hand, none of the five examined Japanese herbal medicines displayed ERα estrogenic activity in the yeast two-hybrid assay (data not shown).

The three herbal medicines that showed estrogenic activity in the yeast two-hybrid assay had their estrogenic activity assessed after being metabolized. The herbal medicines were pre-treated with a rat liver S9 mixture that included metabolic enzymes, and their estrogenic activity was compared with the estrogenic activity of the unmetabolized herbal medicines using the yeast two-hybrid assay. The activity levels of the metabolized products are shown in Fig. 2b. The three herbal medicines that exhibited estrogenic activity in the yeast two-hybrid assay also showed significantly elevated ERβ-dependent estrogenic activity after being treated with the rat liver S9 fraction. These results showed that metabolizing *UKT*, *KSS*, and *NSS* with the rat liver S9 fraction increased their ERβ-dependent estrogenic activity. On the other hand, no significant differences between the ERα-dependent estrogenic activity of the metabolized and unmetabolized medicines were seen for any of the five examined Japanese herbal medicines (data not shown).

### Estrogenic activity of the crude drugs found in *UKT*

In order to clarify which crude drugs were responsible for the pharmacological activity of the herbal medicines, we focused on *UKT*. As shown above, both *UKT* and its metabolites exhibited strong estrogenic activity in both the cell proliferation bioassay and the yeast two-hybrid assay. Therefore, we investigated the estrogenic activity of the crude drugs present within *UKT* using the cell proliferation bioassay and yeast two-hybrid assay.

**Fig. 3:**
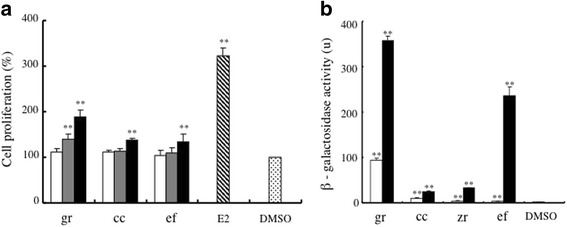
The estrogenic activity of the crude drugs present in *UKT.* **a** MtT/Se cells were incubated with each of the crude drugs found in *UKT* at a concentration of 1 μg/ml (white columns), 5 μg/ml (gray columns), or 10 μg/ml (black columns), and the cell proliferation induced by treatment with these crude drugs or 10^− 7^ M of E2 for 6 days was evaluated. Cell proliferative activity is shown as a percentage compared with that induced by DMSO alone in the MTT assay. **significantly increased compared with the activity induced by DMSO, *p* < 0.01. **b** The β-galactosidase activity of all 12 of the crude drugs present in *UKT* was evaluated at concentrations of 0.1 mg/ml (white columns) and 0.5 mg/ml (black columns). The data for the crude drugs that did not display significant activity in the yeast two-hybrid assay are not shown. **significantly increased compared with the activity of DMSO, *p* < 0.01

In the cell proliferation bioassay, it was found that glycyrrhizae radix, cinnamomi cortex, and evodiae fructus all induced significantly greater cell proliferation than DMSO at a final concentration of 10 μg/ml (*p* < 0.01, Fig. 3a). Among these three crude drugs, glycyrrhizae radix also induced significantly greater proliferative activity than DMSO at a concentration of 5 μg/ml. We also investigated the ERβ-dependent estrogenic activity of the crude drugs present within *UKT* using the yeast two-hybrid assay. As a result, glycyrrhizae radix, cinnamomi cortex, zingiberis rhizoma, and evodiae fructus were found to exhibit significantly greater activity than DMSO at final concentrations of 0.5 mg/ml and 0.1 mg/ml (Fig. 3b). The activity levels of glycyrrhizae radix and evodiae fructus remained high at a concentration of 0.5 mg/ml, and glycyrrhizae radix even demonstrated strong activity at a concentration of 0.1 mg/ml. None of the other crude drugs displayed significant estrogenic activity in the cell proliferation bioassay or yeast two-hybrid assay. These results suggest that the strong estrogenic activity of *UKT* can be attributed to the crude drugs it contains; i.e., glycyrrhizae radix, cinnamomi cortex, zingiberis rhizoma, and evodiae fructus, especially glycyrrhizae radix.

### Synergistic effects of the crude drugs during combination treatment

**Fig. 4:**
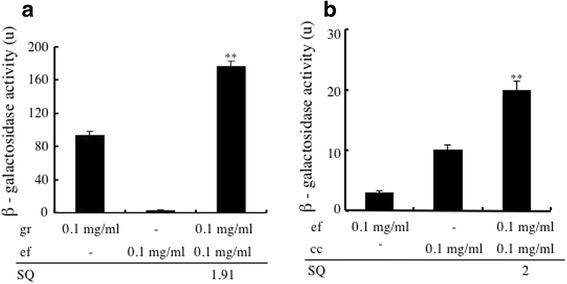
Synergistic effects between the crude drugs found in *UKT* according to the yeast two-hybrid assay. **a** The synergistic effects of combining glycyrrhizae radix with other crude drugs on ERβ-dependent estrogenic activity. **b** The synergistic effects of combining evodiae fructus with other crude drugs on ERβ-dependent estrogenic activity. The β-galactosidase activity of the crude drugs alone and various pairs of crude drugs were evaluated at concentrations of 0.1 mg/ml (black columns). **significantly increased compared with the sum of the activity levels of the two crude drugs alone, *p* < 0.01. SQ values were calculated to determine whether each combination had synergistic effects, as described in the materials and methods. The data for the combinations that did not have significant synergistic effects in the yeast two-hybrid assay are not shown

As described above, glycyrrhizae radix exhibited the strongest estrogenic activity of all the crude drugs in *UKT* and is present in all three of the Japanese herbal medicines that demonstrated estrogenic activity in the MtT/Se cell proliferation bioassay and ERβ-dependent estrogenic activity in the yeast two-hybrid assay; i.e., *UKT*, *KSS*, and *NSS*. In order to detect inter-crude drug synergistic effects on ERβ-dependent estrogenic activity, the combined effects of 0.1 mg/ml glycyrrhizae radix and 0.1 mg/ml of one of the other crude drugs present in *UKT* were examined, and then SQ were calculated. The β-galactosidase activity of glycyrrhizae radix, evodiae fructus, and glycyrrhizae radix+evodiae fructus are shown in Fig. 4a, and the SQ for glycyrrhizae radix+evodiae fructus was calculated according to the method described above. The activity of glycyrrhizae radix+evodiae fructus was significantly greater than the sum of the activity levels of these two crude drugs alone (*p* < 0.01), which resulted in it having an SQ of 1.91 (Fig. 4a). Furthermore, we examined the contribution of such synergistic effects to ERβ-dependent estrogenic activity by combining evodiae fructus with other crude drugs. Accordingly, the β-galactosidase activity of evodiae fructus, cinnamomi cortex, and evodiae fructus+cinnamomi cortex were assessed, and we found that evodiae fructus+cinnamomi cortex exhibited significantly greater activity than the sum of the activity levels of evodiae fructus and cinnamomi cortex alone (*p* < 0.01). The evodiae fructus+cinnamomi cortex combination had an SQ of 2.0 (Fig. 4b). We continued examining the contribution of such synergistic effects to ERβ-dependent estrogenic activity by combining cinnamomi cortex with the other crude drugs present in *UKT*. However, no other synergistic effects were found. These findings suggest that the glycyrrhizae radix+evodiae fructus and evodiae fructus+cinnamomi cortex combinations have synergistic effects on the estrogenic activity of *UKT*.

## Discussion

Japanese herbal medicines are often prescribed for menopausal syndrome, which is caused by the cessation of both egg maturation and sex hormone (principally estrogen and progesterone) secretion. Therefore, we examined the pharmacological activity of Japanese herbal medicines that are used to treat menopausal syndrome, especially their estrogenic activity. In this study, the estrogenic activity of Japanese herbal medicines was evaluated using a combination of an ER-dependent cell proliferation bioassay and an ER-dependent reporter assay. ER-dependent bioassays based on MtT/Se cells, which express receptors for both endogenous ERα and ERβ [16], can be utilized to determine the overall effects of signaling mediated by these two receptors. In order to clarify the estrogenic responses initiated by ERα or ERβ, we used an ER-dependent yeast two-hybrid reporter assay. As described in the results, *UKT*, *KSS*, and *NSS* increased the proliferation of MtT/Se cells, and this activity was markedly inhibited by ICI, an ER antagonist. These results suggest that the effects of *UKT*, *KSS*, and *NSS* on the proliferation of MtT/Se cells are directly mediated by the ER.

On the other hand, in the yeast two-hybrid assay *UKT*, *KSS*, and *NSS* exhibited ERβ-dependent estrogenic activity. Furthermore, a previous study involving a luciferase assay also showed that *UKT* and *KSS* displayed such activity [22]. However, Watanabe et al. reported that they could not detect any agonistic activity of *UKT* or *NSS* towards hER [23], but they used aqueous solutions of herbal medicines. As E2 is soluble in fat, we extracted the herbal medicines using ethanol and prepared DMSO solutions of the medicines. Thus, the different extraction solvents used might explain the abovementioned discrepancies.

In a previous study, Pueraria mirifica, a phytoestrogen-rich traditional herbal plant, bound more strongly to ERβ than ERα [18], and a herbal medicine composed of 18 different crude drugs displayed slightly higher affinity for ERβ [24]. *UKT*, *KSS*, and *NSS* are mixtures of various traditional herbal plants, and therefore, we examined whether these Japanese herbal medicines are also rich in phytoestrogens.

**Fig. 5:**
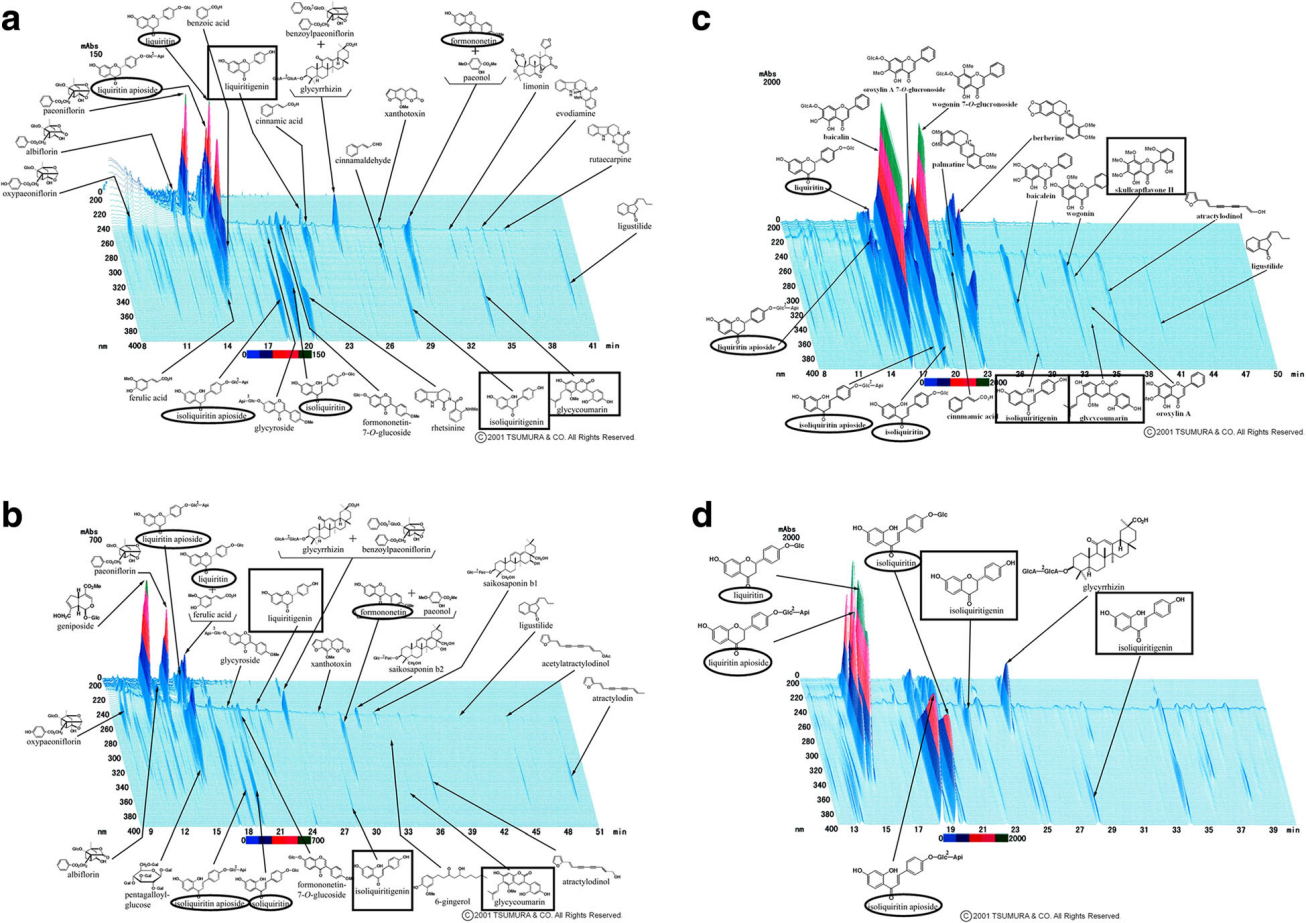
3D-HPLC patterns of three Japanese herbal medicines and glycyrrhizae radix. The 3D-HPLC patterns of *UKT* (**a**), *KSS* (**b**), *NSS* (**c**), and glycyrrhizae radix (**d**) are shown. The constituents in the quadratic boxes possess phenolic and hydroxyl moieties, and those in the oval boxes might be convertible into active estrogenic constituents; i.e., liquiritigenin or isoliquiritigenin, through metabolic reactions

Phytoestrogens are naturally occurring chemicals of plant origin, which have the ability to cause estrogenic and/or antiestrogenic effects due to their structural similarity to the human hormone E2 [25]. The most common phytoestrogens can be divided into four kinds of compound, isoflavones, coumestans, prenylflavonoids, and lignans, all of which possess phenolic and hydroxyl moieties [25]. As was shown in the 3D-HPLC patterns provided by the Tsumura Corporation, isoliquiritigenin and glycycoumarin, which were detected in all three of the Japanese herbal medicines that exhibited estrogenic activity in the present study (*UKT*, *KSS*, and *NSS*); liquiritigenin, which was detected in *UKT* and *KSS*; and skullcapflavone II, which was only detected in *NSS*, also possess phenolic and hydroxyl moieties (Fig. 5). Liquiritigenin and isoliquiritigenin have been found in the licorice species *Glycyrrhiza glabra*, Glycyrrhiza uralensis, and Glycyrrhiza inflata [26] and licorice root extracts that displayed estrogenic activity [27]. The isoliquiritigenin found in *Glycyrrhiza glabra* exhibits stronger docking energy when it binds to ERβ than when it binds to ERα [28]. In addition, previous studies have reported that liquiritigenin demonstrated greater selective affinity for ERβ than for ERα [29–31]. Isoliquiritigenin is the precursor chalcone of liquiritigenin, and a previous study detected a significant reduction in isoliquiritigenin content and a corresponding increase in liquiritigenin formation in cell-based assays, which suggested that the conversion of isoliquiritigenin to liquiritigenin via cyclization might occur under physiological conditions [30]. Glycycoumarin, which was detected in *UKT*, *KSS*, and *NSS*, is a member of the coumarin family that exhibits estrogenic activity [32], and it has also been found to be an estrogen agonist [33]. As shown in the abovementioned 3D-HPLC analysis, the structure of glycycoumarin is similar to that of genistein, which has a linear and polar structure. ERβ has a flexible binding pocket [34], and it can accept a linear group and tolerate the resultant polarity [35]. Therefore, glycycoumarin might also have greater selective affinity for ERβ. Little is known about the estrogenic activity of skullcapflavone II, which is present in *NSS*, but skullcapflavone II is a member of the flavone family, which is the most common type of phytoestrogen. Based on the above points, it is suggested that the estrogenic activity of *UKT*, *KSS*, and *NSS* is due to constituents with common phytoestrogen structures. In the present study, these Japanese herbal medicines demonstrated strong ERβ-dependent estrogenic activity, but none of them displayed ERα estrogenic activity in the yeast two-hybrid assay.

**Fig. 6:**
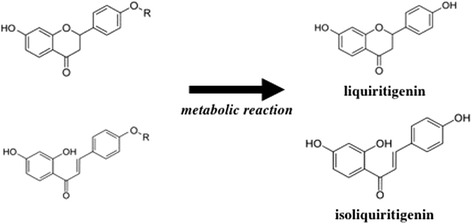
A proposed metabolic pathway. R: a sugar group or a methyl group

We also investigated the estrogenic activity of Japanese herbal medicines after they had been metabolized using the rat liver S9 fraction. *UKT*, *KSS*, and *NSS* exhibited greater ERβ-dependent estrogenic activity after they had been metabolized. Due to the very low abundance and great variety of Japanese herbal medicine metabolites, we did not identify the metabolites produced by treating the herbal medicines with the rat liver S9 fraction using 3D-HPLC analysis in the current study. However, as shown in the abovementioned 3D-HPLC patterns of *UKT*, *KSS*, and *NSS*, the structures of the constituents within the oval boxes were based on a sugar or a methyl group bound to a liquiritigenin or an isoliquiritigenin. It might be possible to convert these structures into liquiritigenin or isoliquiritigenin through previously proposed metabolic pathways [36–38] (Fig. 6), and as described above liquiritigenin and isoliquiritigenin have been shown to be active estrogenic constituents. Therefore, this provides a possible explanation for the increases in the estrogenic activity of *UKT*, *KSS*, and *NSS* seen after they were treated with the S9 fraction. On the other hand, *TSS*, *KBG*, and their metabolites, which do not contain the most common phytoestrogen structures (data not shown), did not exhibit any estrogenic activity in the MtT/Se cell proliferation bioassay or the yeast two-hybrid assay. Thus, we consider that these two herbal medicines might exert pharmacological effects against menopausal syndrome via other mechanisms.

Herbal medicines are mixtures of several crude drugs. In order to clarify which crude drugs were responsible for the pharmacological activity of the examined herbal medicines, we focused on *UKT* and investigated the estrogenic activity of the crude drugs present within it and the synergistic effects of pairs of these crude drugs. The proportions of the crude drugs in *UKT* ranged from 3.7% to 15% (Table 1). Therefore, in this study we evaluated the estrogenic activity of the crude drugs at 10% of the tested concentrations of the Japanese herbal medicines, and glycyrrhizae radix exhibited the strongest estrogenic activity of all the crude drugs found in *UKT*.

As discussed above, *UKT*, *KSS*, *NSS* and their metabolites possess some constituents with common phytoestrogen structures. We found that these constituents were mostly derived from the common crude drug glycyrrhizae radix (Fig. 5), which is a rich source of flavonoids [39, 40], and previous studies have shown that it exhibits estrogenic activity [14, 27, 41]. Furthermore, other crude drugs (cinnamomi cortex, evodiae fructus, and zingiberis rhizoma) displayed ERβ-dependent estrogenic activity. It was reported that cinnamomi cortex demonstrated anti-allodynic activity [42]; evodiae fructus suppressed the proliferation of human epithelial ovarian cancer cells [43]; and zingiberis rhizoma induced nitric oxide production, which inhibited tumor cell growth [44]. As far as we know, this was the first study to examine the estrogenic activity of these crude drugs. During the analysis of the main constituents of these crude drugs, we did not detect common phytoestrogen structures; i.e., phenolic or hydroxyl moieties. A previous review examined ERβ ligands [45], and some of the ligands did have not phenolic or hydroxyl moieties. Therefore, we speculate that previously unknown phytoestrogen constituents that do not have common phytoestrogen structures, but possess affinity for ERβ, are present in cinnamomi cortex, evodiae fructus, and zingiberis rhizoma. However, zingiberis rhizoma did not increase the proliferation of MtT/Se cells in the current study.

The in vivo activity of estrogen was investigated in a previous review, which demonstrated that it involves an array of factors and is controlled by complex signaling pathways [46]. In the present study, an MtT/Se cell bioassay was used to investigate the overall effects of herbal medicines, which might also involve complex signaling pathways. Zingiberis rhizoma showed estrogenic activity, but did not have estrogenic effects on MtT/Se cells.

This study is the first to detect inter-crude drug synergistic effects on ERβ-dependent estrogenic activity. Herbal medicines are made from raw plant materials, and their compositions are complex. In addition, even if the same crude drugs are present in different herbal medicines their ratios can vary. Therefore, we combined pairs of crude drugs in 1:1 ratios. This is an easy way of comparing the effects of combinations of crude drugs. We found that evodiae fructus had synergistic effects when combined with glycyrrhizae radix, and evodiae fructus also had synergistic effects when combined with cinnamomi cortex. Evodiae fructus exhibited 78-fold higher activity when its concentration was increased 5-fold. Hence, in addition to the classical ligand-binding pattern in which one ligand binds to one LBD [34, 38, 47], we speculate that one ligand can bind to more than one LBD through a bridge-bond effect. Such mechanisms could be involved in the abovementioned synergistic effects. However, this issue needs to be clarified in future studies. In addition, several reports have shown that the unliganded ER may be transcriptionally activated by certain post-translational modifications [48]. In MCF-7 breast cancer cells, it was shown that the phosphorylation of a kinase was sufficient to allow it to directly bind to ER and activate them [49]. This mechanism might also be responsible for some synergistic effects. The combination of crude drugs can affect cell homeostasis, activate some unliganded ER, and have synergistic effects. Synergistic effects might explain why herbal medicines, which have been prescribed for thousands of years, work better than the administration of their crude drug constituents alone.

## Conclusions

*UKT*, *KSS*, and *NSS* exhibited estrogenic activity in this study, and their activity increased further after they were metabolized. Such activity might be the key to their efficacy against menopausal syndrome. Glycyrrhizae radix is a crude drug that is found in all three of these Japanese herbal medicines, and it also displayed strong estrogenic activity. By analyzing the main constituents of these three herbal medicines, we found that some of their constituents possessed common phytoestrogen structures; i.e., phenolic and hydroxyl moieties. In addition, we demonstrated for the first time that cinnamomi cortex, evodiae fructus, and zingiberis rhizoma all display ERβ-dependent estrogenic activity. The abovementioned crude drugs might contain some new phytoestrogen constituents that do not possess common phytoestrogen structures, but exhibit affinity for ERβ. We also identified synergistic effects between evodiae fructus and glycyrrhizae radix, and between evodiae fructus and cinnamomi cortex for the first time. Synergistic effects between crude drugs increase the efficacy of herbal medicines. However, the mechanisms responsible for these effects remain unknown.

## Abbreviations

DMEM: Dulbecco’s modified Eagle’s medium
DMSO: dimethyl sulfoxide
E2: 17β-estradiol
ER: estrogen receptor
FBS: fetal bovine serum
HPLC: high-performance liquid chromatography
HS: horse serum
LBD: ligand-binding domain
ONPG: o-nitrophenyl-β-D-galactopyranoside
SQ: synergism quotient
TIF2: transcriptional intermediary factor 2
